# Loading Recommendations for Muscle Strength, Hypertrophy, and Local Endurance: A Re-Examination of the Repetition Continuum

**DOI:** 10.3390/sports9020032

**Published:** 2021-02-22

**Authors:** Brad J. Schoenfeld, Jozo Grgic, Derrick W. Van Every, Daniel L. Plotkin

**Affiliations:** 1 Department of Health Sciences, CUNY Lehman College, Bronx, NY 10468, USA; vaneverd@uwindsor.ca (D.W.V.E.); danielplotkin96@gmail.com (D.L.P.); 2 Institute for Health and Sport, Victoria University, Melbourne, VIC 8001, Australia; jozo990@hotmail.com

**Keywords:** high-load, low-load, strength, hypertrophy, muscular endurance

## Abstract

Loading recommendations for resistance training are typically prescribed along what has come to be known as the “repetition continuum”, which proposes that the number of repetitions performed at a given magnitude of load will result in specific adaptations. Specifically, the theory postulates that heavy load training optimizes increases maximal strength, moderate load training optimizes increases muscle hypertrophy, and low-load training optimizes increases local muscular endurance. However, despite the widespread acceptance of this theory, current research fails to support some of its underlying presumptions. Based on the emerging evidence, we propose a new paradigm whereby muscular adaptations can be obtained, and in some cases optimized, across a wide spectrum of loading zones. The nuances and implications of this paradigm are discussed herein.

## 1. Introduction

Resistance training (RT) is well-established as an effective interventional strategy to enhance muscular adaptations. These adaptations include, but are not limited to, increases in muscle strength, size, and local muscular endurance. Evidence indicates that optimizing these adaptations requires manipulation of RT variables [1,2]. The magnitude of load, or amount of weight lifted in a set, is widely considered one of the most important of these variables. Evidence indicates that alterations in training load can influence the acute metabolic, hormonal, neural, and cardiovascular responses to training [1]. How these acute responses translate into long-term adaptations remains somewhat contentious.

**Figure 1:**
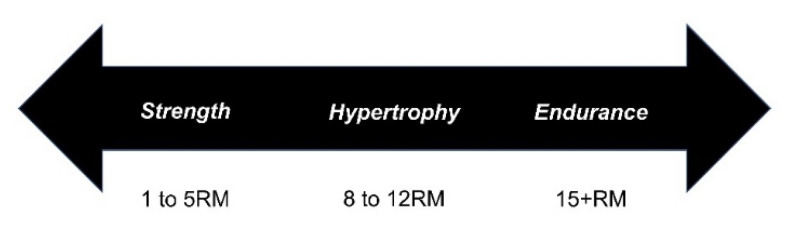
Schematic of the repetition continuum proposing that muscular adaptations are obtained in a load-specific manner. Repetition maximum (RM).

Loading recommendations are typically prescribed along what has come to be known as the “repetition continuum,” also known as the “strength-endurance continuum” [3] (see Figure 1). The repetition continuum proposes that the number of repetitions performed at a given magnitude of load will result in specific adaptations as follows:

1. A low repetition scheme with heavy loads (from 1 to 5 repetitions per set with 80% to 100% of 1-repetition maximum (1RM)) optimizes strength increases.
2. A moderate repetition scheme with moderate loads (from 8 to 12 repetitions per set with 60% to 80% of 1RM) optimizes hypertrophic gains.
3. A high repetition scheme with light loads (15+ repetitions per set with loads below 60% of 1RM) optimizes local muscular endurance improvements.

Support for the repetition continuum is derived from the seminal work of DeLorme [4], who proposed that high-load resistance exercise enhances muscle strength/power while low-resistance exercise improves muscular endurance, and that these loading zones are incapable of eliciting adaptations achieved by the other. Subsequent research by Anderson and Kearney from 1982 [5] and Stone et al., 1994 [6] provided, in part, additional support to Delorme’s hypothesis, forming the basis of what is now commonly accepted as theory. However, emerging research challenges various aspects of the theory. The purpose of this paper is to critically scrutinize the research on the repetition continuum, highlight gaps in the current literature, and draw practical conclusions for exercise prescription. Based on the evidence, we propose a new paradigm whereby muscular adaptations can be obtained, and in some cases optimized, across a wide spectrum of loading zones. The nuances and implications of this paradigm are discussed herein.

## 2. Strength

Strength can be broadly defined as the ability to produce maximum force against an external resistance [7]. The leftward aspect of the repetition continuum has been referred to as the “strength zone” (see Figure 1), indicating optimum gains in this parameter are attained by the performance of 1 to 5 repetitions per set. It is theorized that training in the “strength zone” enhances neuromuscular adaptations that facilitate force production [3]. In support of this theory, Jenkins et al. [8] demonstrated greater increases in percent voluntary muscle activation and electromyographic amplitude when performing leg extension RT to failure with 80% 1RM compared to 30% 1RM over a 6-week study period. Psychological factors are believed to be involved as well, as repeated heavy load lifting may help lifters acclimate to exerting a maximal effort; however, the psychological contribution to strength-related adaptations remains equivocal [9].

Strength is most commonly assessed via 1RM testing that involves the performance of dynamic constant external resistance exercise using either free weights or exercise machines. Meta-analytic data of this metric shows a clear advantage to using heavier compared to lighter loads when the number of sets are similar between conditions. For example, a recent meta-analysis [10] reported a moderate to large effect size (ES) difference (ES = 0.58) favoring high- (>60% 1RM) vs. low- (≤60% 1RM) load training based on pooled data from 14 included studies. Results held true independent of whether testing was conducted in exercises for the upper or lower body. A meta-analysis by Csapo et al. [11] reported similar results in older individuals, with an overall pooled effect size difference (ES = 0.43) that indicated a moderate magnitude of effect in favor of heavy load training. Importantly, all included studies showed a strength-related advantage to using high- compared to low loads (i.e., effect sizes from all studies resided in the “favors high-load” side of the forest plot).

The strength-related benefits of heavier loads are generally observed independent of RT volume, whether expressed as the number of sets performed or the total work performed, commonly termed “volume load” (sets × repetitions × load). This is an important point of note as heavier load training necessarily results in fewer repetitions performed on a set-equated basis compared to light loads. Thus, it can be inferred that load is the dominant variable for increasing 1RM, with other variables seemingly of secondary consequence [12]. 

It should be noted that while heavy load training is clearly requisite for maximizing 1RM, significant strength gains in this test are routinely observed with the use of low-loads (≥20 repetitions per set) [13,14,15]. Even resistance-trained individuals show increases in strength when training with very light loads, albeit to a lesser extent than with the use of heavy loads [16,17]. Research in highly trained individuals is lacking on the topic, but it seems likely that continued maximum strength improvements become increasingly dependent on training closer to a person’s 1RM as one approaches their genetic ceiling. Indeed, evidence indicates that the principle of specificity (also known as specific adaptation to imposed demands) becomes more relevant based on one’s level of training experience [18]. Further study is warranted in elite athletes to better understand how training experience impacts the acquisition of strength with respect to the magnitude of load.

Research comparing different loading strategies tends to support a dose–response relationship between load and strength gains. Multiple studies have reported greater 1RM improvements when training in the so-called “strength zone” (1 to 5 repetitions) vs. the “hypertrophy zone” (8 to 12 repetitions) [19,20,21,22], although these findings are not universal [23,24]. Discrepancies between studies remain unclear, but it appears the dose–response relationship is more pronounced in resistance-trained individuals. It is not clear whether regularly training with maximal loads promotes a superior strength-related response on this metric and, if so, how such loading should be integrated into a comprehensive training program to optimize results.

When considered in total, the literature does seem to support the existence of a “strength zone” for increasing 1RM, consistent with the concept of a repetition continuum. The apparent dose–response relationship provides further evidence for the causality of the adaptation. Some researchers have proposed that the periodic “practice” of lifting with heavy loads is sufficient to maximize strength adaptations [16,25], but this hypothesis remains speculative. Further research is necessary to determine how frequently one needs to lift in the leftward portion of the repetition continuum to elicit maximal 1RM increases.

An important point to consider is that researchers generally carry out 1RM testing on exercises performed as part of the interventional program. This necessarily biases results in favor of heavier lifting protocols, as the training itself is highly specific to the testing modality. Indeed, the advantage of heavy load training on strength-related measures dissipates when testing is carried out on a modality different than that used in the study training program. The aforementioned meta-analysis by Schoenfeld et al. [10] showed a small, statistically non-significant benefit (ES = 0.16) to the use of heavier loads when testing on an isometric device; our recent original study on the topic further supports this finding [26]. There was a lack of sufficient data at the time to subanalyze isokinetic strength with meta-regression, but the findings from available evidence are conflicting; some studies show a benefit of heavy load training [27,28], others show a benefit to low-load training [29] and yet others show no differences between conditions on this metric [30,31]. The reason for these incongruities is uncertain and warrant further investigation. 

Although testing on a neutral device (e.g., isometric dynamometer) suggests that the magnitude of load may not influence strength-related adaptations, the question remains as to whether this has meaningful implications from a practical standpoint. Such testing generally isolates strength assessment to a single joint (e.g., knee extensors, elbow flexors, etc.). However, strength is most often applied as the coordinated effort of multiple joints in the performance of functional activities. Thus, it remains speculative as to how results from isometric/isokinetic assessments translate to athletic performance or the ability to carry out tasks of everyday living. The topic warrants further investigation. A summary of studies on the topic is presented in Table 1.

## 3. Hypertrophy

Muscle hypertrophy refers to the growth of muscle tissue, which can manifest in a variety of ultrastructural adaptations [32]. The mid-range of the repetition continuum (from 8 to 12 repetitions) is commonly referred to as the “hypertrophy zone” [33], reflecting the belief that such a loading scheme is ideal for building muscle (see Figure 1). The practical implications of this viewpoint are highlighted in the American College of Sports Medicine RT guidelines, whereby the use of moderate loads is recommended for hypertrophy training [2]. Other research papers provide similar loading recommendations when training to maximize muscle development [1,34].

The concept of a hypertrophy zone is consistent with anecdotal evidence that bodybuilders generally train with moderate loads [35]. Research-based support for the “hypertrophy zone” comes largely from acute studies showing greater post-exercise elevations in anabolic hormones when training in a moderate repetition range [36]. However, the relevance of transient exercise-induced systemic hormonal spikes on muscular adaptations remains dubious [36], thus calling into question the basis of this rationale. That said, several alternative lines of empirical evidence can be used to draw objective conclusions on the effects of the magnitude of load on muscle growth.

When attempting to draw inferences on the topic, one line of evidence to evaluate is the acute molecular and muscle protein synthetic (MPS) response to an exercise bout at differing loading zones. In this regard, research on the topic has produced somewhat discrepant results. Some studies show an impaired acute MPS response when training with lower loads [37,38] while others report similar increases in mixed and myofibrillar protein synthesis rates [39]. Other research demonstrates divergent responses in intracellular anabolic signaling and myogenic gene expression when training in moderate- (from 74% to 85% 1RM) and lower (from 54% to 65% 1RM) loading zones, with selective activation of different kinase pathways observed between conditions [40,41]. 

When attempting to reconcile the acute data, level of effort appears to be an explanatory variable accounting for discrepancies in results. Specifically, studies showing an impairment in the anabolic response with light loads employed work-matched protocols whereby participants stopped the low-load sets well short of fatigue [37,38]. This is notable given research indicating that training with a high level of effort is particularly critical for maximizing hypertrophic adaptations in low-load training [42]. Consistent with this line of evidence, research where participants expended a high level of effort suggests that the MPS response to low-load training is at least as robust as when training with heavier loads [39]. That said, preliminary evidence for potential differences in intracellular anabolic signaling between loading zones cannot be discounted [40,41], and may have practical implications for RT program design. However, while acute studies on intracellular signaling and MPS are beneficial for understanding mechanisms and generating hypotheses for applied implications, results may not necessarily replicate over successive exercise trials. Indeed, evidence shows a lack of correlation between acute post-exercise MPS measures and chronic increases in muscle mass [43]. Hence, an examination of longitudinal data is necessary to provide insights into long-term term hypertrophic adaptations.

Early evidence from longitudinal studies suggested that light-load training produced suboptimal skeletal muscle hypertrophy. A 2007 review of the literature by Wernbom et al. [44] concluded a hypertrophic benefit to training with loads >60% 1RM. However, the conclusion was based on a limited amount of data that directly compared the effects of training with varying loads on muscle hypertrophy at that point in time. Multiple studies have subsequently been published on the topic, with the vast majority indicating similar hypertrophy across a wide spectrum of loading ranges. The aforementioned meta-analysis by Schoenfeld et al. [10] found no difference in whole muscle hypertrophy between studies comparing high loads (>60% 1RM) versus low loads (<60% 1RM). The trivial effect size difference (0.03) and relatively narrow 95% confidence intervals (−0.16 to 0.22) reinforce the lack of relevance of loading as a standalone variable for hypertrophic outcomes. Moreover, sub-analysis found these results held true independent of body region (i.e., upper and lower body musculature). 

From an age-related standpoint, light load training appears to be at least as effective as heavier load training, if not more so, for inducing hypertrophy in older individuals. Meta-analytic data from Straight et al. [45] found that while older individuals responded to both higher and lower loading protocols, muscle hypertrophy was attenuated in type II muscle fibers when training with heavier loads; the difference between loading strategies explained ~15% of the variance in change in fiber size. A mechanistic explanation for these findings is not clear, but conceivably may be related to age-associated difficulty training with heavier loads due to joint-related conditions (e.g., osteoarthritis).

Although a majority of studies on the topic have been carried out in untrained participants, the available evidence indicates that findings hold true in those with RT experience. For example, our group [17] reported similar increases in muscle thickness of the biceps, triceps and quadriceps between moderate- (~10RM) and light- (~30RM) loads in a cohort of resistance-trained men performing a total body RT program over 8 weeks. Likewise, Morton et al. [16] found that training with 8 to 12RM vs. 20 to 25RM produced significant changes in lean body mass and type I and type II muscle fiber cross-sectional area (CSA) of the vastus lateralis in a group of resistance-trained men following a 12-week total body training program, with no observed differences between the groups.

The effects of volume must be taken into consideration when interpreting data on hypertrophic loading outcomes. Volume, expressed as the number of sets performed, is an important driver of muscle hypertrophy, with an established linear dose-response relationship [46]. However, some researchers have postulated that volume load may be the best metric for assessing exercise-induced hypertrophic changes [47]. On a set-equated basis, lighter load sets would necessarily result in greater volume loads compared to heavier loads due to the higher number of repetitions performed, therefore potentially influencing results.

Few studies have investigated the hypertrophic effects of high- versus low-loads when equating for volume load. Lopes et al. [48] found no differences in fat-free mass between volume load-equated low (3 sets of 20RM) and high (6 sets of 10RM) load training protocols in resistance-trained men who performed a total-body RT program for 6 weeks. These results must be interpreted with caution as body composition measures were obtained by skinfold analysis, and thus may not necessarily reflect changes in muscle mass. Alternatively, Holm et al. [49] reported a significantly greater increase in muscle CSA when training at 70% vs. 15% of 1RM on a volume load equated basis in a cohort of untrained young men over a 12-week study period. However, the low-load condition terminated sets far short of muscle failure, confounding the ability to draw relevant inferences. Given the paucity of well-designed studies on the matter, further research is warranted to determine how volume load may affect muscle growth with different loading schemes. 

Although a majority of published research has focused on comparing moderate versus light load training, several studies have investigated potential differences in heavy- vs. moderate-load protocols. Our group [20] randomized resistance-trained men to perform volume load-equated RT using either a bodybuilding-type protocol (3 sets of ~10RM) or a powerlifting-type protocol (7 sets of ~3RM). Training was carried out 3 times per week for 8 weeks. While results showed similar increases in biceps brachii muscle thickness between conditions, participants in the powerlifting-type group displayed signs of overtraining and joint-related issues at study’s end whereas no such symptoms were observed in the bodybuilding-type group. The findings suggest that although hypertrophy can be achieved using either heavy or moderate loads on a work-matched basis, heavy load protocols may not be sustainable for maximizing muscle growth due to negative consequences of the higher training volumes. In a related study, Klemp et al. [24] randomized resistance-trained men to perform the squat and bench press 3 times per week at a loading range of either 8 to 12 repetitions or 2 to 6 repetitions in a daily undulating periodized fashion. After the 8-week interventional period, similar changes in muscle thickness were observed between conditions for the pectoralis major and quadriceps. The results lend support to the theory that heavy loading can be an effective means to increase hypertrophy when combined with higher volume loads. 

The data for hypertrophy are more equivocal in studies equating the number of sets between high- and moderate-load protocols. Our group [21] found greater increases in muscle thickness of the lateral thigh when resistance-trained men performed 3 sets of 8 to 12RM compared to 2 to 4RM. Conversely, Mangine et al. [22] reported similar changes in muscle thickness between high- and moderate-load training in a cohort of resistance-trained men following 8 weeks of total body RT exercise; interestingly, greater gains in dual x-ray absorptiometry-derived lean arm mass were noted for the heavier load group. Discrepancies in findings may be attributed to the fact that the design in Mangine et al. [22] had participants in the heavy load group rest 3 min between sets while those in the moderate load group rested just 1 min. In contrast, all participants in the study by our group [21] rested 2 min between sets. Given research showing a potential hypertrophic detriment to employing short rest intervals in resistance-trained individuals [50,51], it is conceivable that differences in rest periods may have confounded the results of Mangine et al. [22].

Some researchers have proposed that training across rep ranges may induce a fiber type-specific response whereby lower loads promote a preferential increase in hypertrophy of type I fibers and heavier loads favor hypertrophy of type II fibers [52]. Several lines of evidence provide a theoretical basis for this claim. For one, type I fibers are considered “endurance-oriented” fibers, with a high capacity to resist fatigue but relatively low force-producing capacity [53]. Thus, it is conceivable that longer times under tension associated with lighter load training may help to stimulate these fibers to a greater degree than heavier load training. Moreover, the greater acidosis and corresponding accumulation of H^+^ during higher repetition may interfere with calcium binding in type II fibers, thereby placing an even greater burden on type I fibers to maintain force output [54].

Evidence from low-load blood flow restriction (BFR) training lends further theoretical support to a fiber-type specific loading response, with several studies showing preferential hypertrophy of type I fibers [55,56,57]. Although low-load BFR training and traditional low-load training have inherent differences, some researchers have called low-load training a “milder form of low-load blood flow restrictive exercise” [58], suggesting the two forms of exercise may induce adaptations through similar mechanistic actions. Indeed, comparable increases in muscle size commonly are seen between traditional low-load training and low-load BFR training when sets are carried out to muscular failure [59,60]. A detailed discussion of the hypertrophic effects of BFR is beyond the scope of this paper; interested readers are referred to recent reviews on the topic [61,62]. 

Despite the seemingly solid logical rationale, results from both acute and longitudinal research comparing fiber type-specific hypertrophy in high- vs. low-load training have been mixed. A compelling body of studies using surface electromyography (EMG) have shown greater muscle activity with the use of high- compared to low loads [63,64,65,66,67]. However, the inherent limitations of surface EMG analysis precludes the ability to draw inferences as to motor unit recruitment [68]. To account for these issues, Muddle et al. [69] employed a decomposition technique that allowed extraction of single motor unit activities from surface EMG when training at high- (70% maximum voluntary isometric contraction) vs. low- (30% maximum voluntary isometric contraction) loads performed to failure. Analysis of the firing trains from more than 4000 motor units in the vastus lateralis muscle showed that heavier loads were required to recruit the full spectrum of higher threshold motor units, although these results varied somewhat between individuals. Conversely, Morton et al. [67] reported that glycogen was similarly depleted following high- (80% 1RM) and low- (30% 1RM) load training in both type I and type II fibers of the vastus lateralis, indicating similar recruitment across the available motor unit pool. Despite these discrepancies, it seems clear from the literature that a substantial percentage of high-threshold motor units are recruited with low-load training to muscle failure; whether recruitment is equal across loading zones remains somewhat equivocal. 

Regarding longitudinal research, some studies show a fiber type specific response [70,71,72] while others do not [16,73,74]. Discrepancies between findings may be due to differences in the level of effort between studies; those showing similar fiber type adaptations were carried out training to volitional failure, whereas those showing preferential fiber type hypertrophy appear to have not trained to failure. As previously noted, evidence indicates that a high level of effort is requisite for achieving gains in low load training [42], and this may be due to fully stimulating the highest threshold motor units. Interestingly, two studies actually show greater hypertrophy in both type I and type II fibers when training with heavier loads [19,75]. These results appear counterintuitive given the seemingly incontrovertible evidence that whole muscle hypertrophy is similar irrespective of the magnitude of load; if hypertrophy is in fact greater across fiber types when training at heavier vs. lighter loads, what would explain the consistently similar findings in magnetic resonance imaging- and ultrasound-derived measures of hypertrophy between conditions?

In an effort to achieve greater clarity on the topic, a recent study compared the effects of loading on the soleus (a muscle with a very high proportion of type I fibers) and gastrocnemius (a muscle with a mixed fiber type) [26]. Employing a within-subject counterbalanced design, participants performed 4 sets of standing and seated plantarflexion exercise twice per week using a heavy load (6 to 10RM) on one leg and light load (20 to 30RM) on the other leg. After 8 weeks, significant increases in muscle thickness were observed for both the soleus and gastrocnemius; the amount of load did not influence the magnitude of gains. These findings cast doubt as to a load-induced effect on fiber type adaptations. However, it should be noted that the study did not directly assess fiber growth via muscle biopsy, limiting the ability to draw definitive conclusions. 

A recent meta-analysis included studies that performed muscle biopsies and compared the effects of low-load vs. high-load on type I and type II muscle fiber CSA with training carried out to muscular failure [76]. The analysis found no significant difference between low-load vs. high-load for type I muscle fiber CSA. While the effects favored high-load training for type II muscle fiber CSA, they were not statistically significant (effect size: 0.30; 95% confidence interval: −0.05, 0.66; *p* = 0.089), possibly because only five studies were included in the analysis. The results highlight the need for future research on the topic. 

While research is compelling that hypertrophy can be attained by training across a wide spectrum of loading ranges, it remains less clear whether a minimum threshold of loading exists for maximizing hypertrophic outcomes. Several recent studies help to clarify this topic. In a within-subject design, Counts et al. [77] allocated untrained men and women to perform elbow flexion using a load of 70% 1RM in one arm while the other arm trained without using an external load (i.e., “no-load” group). The no-load condition required participants to contract their working muscle as hard as possible throughout the full range of motion of each repetition. After the 6-week training period, similar increases in muscle thickness were observed between conditions, leading the authors to conclude that “muscle growth can occur independent of an external load provided there are enough muscle fibers undergoing mechanotransduction’’ Lasevicius et al. [78] also employed a within-subject design to investigate whether a minimum loading threshold exists for hypertrophic gains over a 12-week study period. The researchers had participants train one arm (elbow flexion) and one leg (leg press) with 20% 1RM and the contralateral limb was then randomly allocated for training at either 40%, 60%, or 80% 1RM. The 20% 1RM condition was always trained first in each session, and the number of sets in the alternative condition was then adjusted to match volume load. Results showed similar increases in CSA for the 40%, 60% or 80% 1RM conditions in both the upper and lower limbs. Alternatively, gains in the 20% 1RM condition were approximately half that achieved with the higher loads. These findings are consistent with those of Buckner et al. [79], who reported significantly greater increases in biceps brachii muscle thickness when training at 70% 1RM compared to 15% 1RM in a cohort of untrained men and women after performing 8 weeks of elbow flexion exercise.

Despite the curious findings of Counts et al. [77] showing marked hypertrophy with no-load training (at least over a 6-week intervention), there does appear to be a minimum threshold for loading below which hypertrophic gains are compromised. Given the evidence that training with 30% 1RM produces comparable hypertrophy to that with heavy loads [73], it can be inferred that the minimum threshold is somewhere in the range of 30% 1RM. However, it is important to note that the number of repetitions achieved at a given percentage of 1RM varies widely between individuals and, in addition to involving genetic factors, specific values ultimately will depend on considerations such as modality (free weights vs. machines), area of the body trained (e.g., upper vs. lower), single vs. multi-joint exercises, and perhaps others [1]. Additionally, while it generally seems that the theory proposed in the repetition continuum is not necessarily valid for hypertrophy, training with low-loads tends to produce more discomfort, displeasure, and a higher rating of perceived exertion than training with moderate-to-high loads [80,81]. Therefore, from a practical standpoint, training with moderate loads is likely to be more enjoyable, which might also impact long-term adherence. A summary of studies on the topic is presented in Table 2.

## 4. Muscular Endurance

Local muscular endurance, operationally defined as the ability to resist muscular fatigue when using a submaximal resistance [82], is purported to be best developed at the rightward aspect of the repetition continuum, corresponding to 15+ repetitions (see Figure 1). Proposed adaptations associated with such training have been attributed to an improved buffering and oxidative capacity, an increase in capillarization and mitochondrial density, and enhanced metabolic enzyme activity [3]. 

Muscular endurance can be expressed either on an absolute or relative basis. Absolute muscular endurance involves performing a set with as many repetitions as possible at a fixed load. For example, the National Football League combine employs a bench press test to assess muscular endurance, whereby the athletes lift 225 lbs (102 kg) to muscle failure; the load is independent of the athlete’s weight or absolute strength levels. Alternatively, relative muscular endurance is assessed by lifting a load at a given percentage of 1RM for as many repetitions as possible. Although there is no generally accepted submaximal percentage for relative muscular endurance testing, it most commonly is assessed using loads between 40% and 60% 1RM.

Early work by Anderson and Kearney [5] lent support for the acquisition of muscular endurance along a repetition continuum. These researchers allocated 43 untrained young men to perform bench press training at either a high- (3 sets of 6 to 8RM), medium- (2 sets of 30 to 40RM) or low- (1 set of 100 to 150RM) load over a 9-week study period. Relative endurance was assessed at 40% of each participant’s 1RM and absolute endurance was assessed at ~27 kg for all participants. Results showed that absolute muscular endurance increased by 41% and 39% (an increase of ~15 repetitions) in the low- and medium-load groups, respectively, while the high-load group realized a 28% gain (an increase of ~9 repetitions). Increases in relative muscle endurance were 22% and 28% for the low- and medium-load groups, respectively, compared to a decrease of 7% in the high-load group. Subsequent research by Stone and Coulter [6] found somewhat contradictory results on the topic in a cohort of untrained young women who performed 5 basic exercises (bench press, squat, lat pulldown, triceps pushdown, and arm curl) using either high- (3 sets of 6 to 8RM), medium- (2 sets of 15 to 20RM) or low- (1 set of 30 to 40RM) loads. Muscular endurance testing was carried out for the upper and lower body (bench press and squat) using both absolute and relative assessments. For the absolute assessment, the gains in upper body muscular endurance favored the medium-load condition compared to the high- and low-load conditions (44% vs. 31% and 20%, respectively), inconsistent with the repetition continuum. Alternatively, the lower body muscular endurance assessment showed findings in line with the proposed repetition continuum, with observed increases of 84%, 80%, and 137% for high-, medium-, and low-loads, respectively. Findings in the relative assessment of muscular endurance based on pretest RM were similar to those observed in the absolute assessment. Upper body results showed a U-shaped response, with increases of 58%, 67%, and 54%, for high-, medium-, and low-loads, respectively. On the other hand, lower body muscular endurance favored training with low- (83%) compared to high- (66%) and medium- (61%) loads. These findings suggest that a repetition continuum for local muscular endurance seems more relevant to the lower body than the upper body musculature. Note that the “medium” and “low” load conditions in both the Anderson and Kearney [5] and Stone and Coulter [6] studies employed repetition ranges >15RM, which encompasses the “endurance” aspect of the repetition continuum. Moreover, a progressively fewer number of sets were performed for the lighter load condition in these studies, raising the question as to whether an advantage would have been shown for muscular endurance if sets had been equated rather than volume load. It is not clear as to why there may be load-dependent differences in muscular endurance between the upper and lower limbs, while no such effect is seen with respect to strength or hypertrophy outcomes; further research is required to better understand this apparent phenomenon.

An important methodological consideration when interpreting the evidence is related to the use of pre-intervention or post-intervention 1RM values. Specifically, some studies that evaluated the effects of training with different loading schemes on muscular endurance used the pre-intervention (baseline) 1RM values to determine load for the endurance tests, whereas others used post-intervention values [20,31]. For example, if one group of participants has an average 1RM in the bench press of 100 kg in the pre-intervention testing and we use 60% of the 1RM for the muscular endurance test, the load would be set to 60 kg. However, if the participants increase their 1RM in the post-intervention to 120 kg, then the 60 kg used in the initial testing would now correspond to 50% of the newly established 1RM, and this would naturally change some of the physiological demands of the test. Another option would be to re-adjust the weight in the post-intervention testing according to the newly established 1RM. In our example, 1RM in the post-intervention was 120 kg and the new load for the muscular endurance test would be set to 72 kg. However, a limitation of this approach is that it biases the values in the test towards the low-load condition. As noted previously, high-load training generally results in greater increases in 1RM and would therefore also require higher loads to be used in the muscular endurance assessment [10]. Indeed, our group [20] showed greater improvements in relative upper body muscular endurance (50% 1RM in the bench press) when training with low- (25 to 35RM) compared to moderate- (8 to 12RM) loads. However, post-study testing was based on post-study 1RM, conceivably biasing results in favor of the low-load condition.

Alternatively, studies in which local muscular endurance testing is based on pre-study 1RM tend to refute the benefit of low-load training for this outcome. Jessee et al. [31] showed similar increases in relative muscular endurance assessed at 42.5% of the pre-test 1RM in the unilateral knee extension following performance of an 8-week training program using loads of either 15% or 70% 1RM. More recently, Buckner et al. [79] found that assessing relative muscular endurance using elbow flexion at 42.5% of pre-test 1RM showed no effect of loading between high- (70% 1RM) and low- (15% 1RM) load conditions.

Given the differential findings observed between studies that use pre-intervention vs. post-intervention 1RM values for determining the load in the muscular endurance tests, future studies may consider using both methods of testing. Assessing muscular endurance while using both pre-intervention and post-intervention 1RM values would likely require multiple days of testing, which may present some logistical limitations. Still, such an approach was adopted by the early work of Stone and Coulter [6]. In that study, when using the pre-intervention 1RM values, all groups experienced an increase in muscular endurance. However, when the load was re-adjusted to post-intervention 1RM values, none of the groups experienced an increase in upper-body endurance, and for the lower-body, a significant increase was found in the group that trained with 6-8RM (31%; ~11 repetitions) and in the group that trained with 30–40RM (33%; ~10 repetitions). No significant differences were observed in the group training with 15–20RM, which would negate the theory proposed in the repetition continuum.

Another option is to use a muscular endurance test not influenced by the changes in 1RM values. For example, one study compared the effects of training with 80% 1RM vs. training with 40% of 1RM [29]. The participants performed an isokinetic test that evaluated total work, which was considered a proxy of muscular endurance. In this study, the group training with 40% 1RM experienced a 15% increase in total work, significantly greater than the increase observed in the 80% 1RM group (5%). Thus, the repetition continuum would seem to be valid when total work in an isokinetic task is used to measure muscular endurance. Still, this finding is based only on one study, highlighting the need for future research.

Studies that have compared the effects of heavy- and moderate-load training show similar increases in muscular endurance between the conditions [21,24]. This indicates a lack of a dose–response relationship on the topic. Hence, if there is in fact a load-induced effect on muscular endurance, which remains questionable, it seemingly is limited to the far rightward aspect of the repetition continuum. A summary of studies on the topic is presented in Table 3. 

## 5. Conclusions

**Figure 2:**
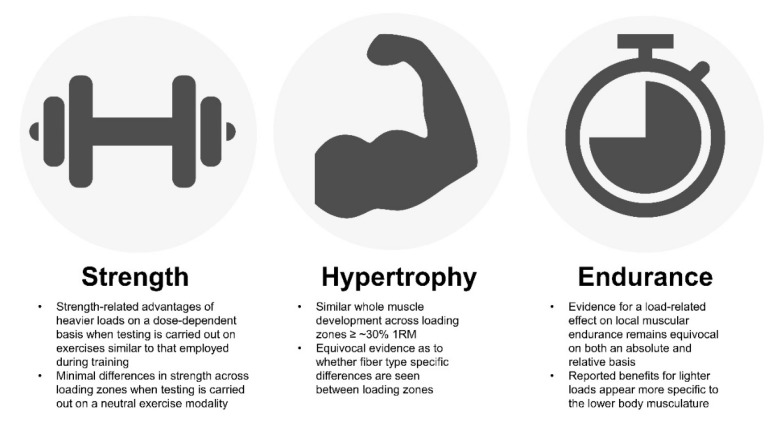
Summary of current evidence on load-specific adaptations from resistance training.

Despite the widespread acceptance of the repetition continuum as a loading paradigm, current research fails to support some of its underlying presumptions. The following evidence-based conclusions can be drawn when taking the body of literature into account, bringing about a new loading paradigm for exercise prescription (see Figure 2). 

Evidence supports the repetition continuum in regard to muscular strength as determined by 1RM testing using dynamic constant resistance exercise. This can be attributed, at least in part, to the fact that testing is customarily carried out on exercises used in the research protocol, which provides a better transfer of training consistent with the principle of specificity. Alternatively, when testing is carried out on an isometric device, there is little difference in strength-related improvements between loading conditions. The practical implications of these findings as they relate to athletic performance and the ability to carry out activities of daily living remain to be determined.

With respect to hypertrophy, the compelling body of literature indicates that similar whole muscle growth (i.e., muscle thickness, CSA) can be achieved across a wide spectrum of loading ranges ≥ ~30% 1RM. These findings are independent of age and training status. Thus, as a matter of principle, there is no ideal “hypertrophy zone.” From a practical standpoint, however, a case can be made that moderate loads provide the most efficient means to achieve muscle development given that light load training involves performing many more repetitions compared to the use of heavier loads, which in turn increases the time spent training. Moreover, the high levels of metabolic acidosis that accompany the use of light loads tends to cause discomfort [81], which in turn can negatively impact adherence. Alternatively, evidence suggests that heavy load training requires more sets to achieve comparable hypertrophy to moderate loads. Not only is this inefficient from a time standpoint, but the combination of heavy loads with high training volumes heightens joint-related stresses and increases the potential for overtraining. Both acute [40,41] and longitudinal [83,84,85,86] data suggest a potential hypertrophic benefit to combining loading ranges as part of a structured RT program, although the practical implications of findings remain questionable; further study is needed to draw stronger conclusions on the topic.

Evidence for a load-specific effect on local muscular endurance remains equivocal. Early work suggested a potential benefit of light load training on muscular endurance, particularly when testing on an absolute basis. That said, the evidence for such an effect is rather weak and seems more relevant to the lower body musculature. Alternatively, research investigating the effects of load on relative muscular endurance is conflicting and, for the most part, does not seem to support recommendations drawn from the repetition continuum.

Overall, there is a paucity of studies carried out in women on the topic. Given evidence that women possess a greater capacity to resist fatigue [87], it is conceivable there may be sex-specific differences in adaptations across the repetition continuum. Future research should endeavor to determine the potential for sexual dimorphism in strength-, hypertrophy-, and endurance-related outcomes when training with different loading schemes.

## Figures and Tables

**Table 1:** Summary of studies that explored the effects of different loads on muscular strength.

Study	Participants	Training Groups (Sets × Load)	Training Duration; Training Frequency	Strength Test	Findings
Aagard et al. 1996 [27]	Young male soccer players (*n* = 17)	4 × 8RM 4 × 16RM 4 × 24RM	12 weeks; 3 times per week	Isokinetic knee extension Isokinetic knee flexion	Knee extension: significantly greater gains in strength in the high load group. Knee flexion: significantly greater gains in strength in the high load group.
Anderson et al. 1982 [5]	Male college students (*n* = 43)	3 × 6–8RM 2 × 30–40RM 1 × 100–150RM	9 weeks; 3 times per week	1RM bench press	Significantly greater gains in strength in the high load group.
Au et al. 2017 [88]	Resistance-trained men (*n* = 32)	3 × 8–12RM 3 × 20–25RM	12 weeks; 4 times per week	1RM bench press 1RM leg press	1RM bench press: significantly greater gains in strength in the high load group. 1RM leg press: no significant between-group differences.
Campos et al. 2002 [19]	Young untrained men (*n* = 27)	4 × 3–5RM 3 × 9–11RM 2 × 20–28RM	8 weeks; 2–3 times per week	1RM squat 1RM leg press 1RM knee extension	1RM squat: significantly greater gains in strength in the high load (3–5 RM) group. 1RM leg press: significantly greater gains in strength in the high load (3–5 RM) group. 1RM knee extension: significantly greater gains in strength in the high load (3–5 RM) group.
Dinyer et al. 2019 [89]	Young untrained women (*n* = 23)	2–3 × 80% 1RM 2–3 × 30% 1RM	12 weeks; 2 times per week	1RM knee extension 1RM shoulder press 1RM knee flexion 1RM lat-pull down	No significant between-group differences in any of the 1RM tests.
Fink et al. 2016 [90]	Young male gymnastics athletes (*n* = 21)	3 × 8–12RM 3 × 30–40RM Mixed high and low load group: 4 weeks of 3 × 8–12RM and 4 weeks of 3 × 30–40RM	8 weeks; 3 times per week	Elbow flexion MVIC	Significantly greater gains in strength in the high load group.
Fink et al. 2016 [91]	Young male gymnastics athletes (*n* = 20)	3 × 8RM 3 × 20RM	8 weeks; 3 times per week	Elbow flexion MVIC	Significantly greater gains in strength in the high load group.
Fisher et al. 2016 [80]	Young recreationally active men (*n* = 7)	3 × 80% MVT 3 × 50% MVT	6 weeks; 1 time per week	Isokinetic knee extension	No significant between-group differences.
Franco et al. 2019 [92]	Undergraduate college women (*n* = 32)	3 × 8–10RM 3 × 30–35RM	9 weeks; 2 times per week	1RM knee extension	No significant between-group differences.
Hisaeda et al. 1996 [30]	Young untrained women (*n* = 11)	8–9 × 4–6RM 5–6 × 15–20RM	8 weeks; 3 times per week	Isokinetic knee extension	No significant between-group differences.
Jenkins et al. 2017 [8]	Young untrained men (*n* = 26)	3 × 80% 1RM 3 × 30% 1RM	6 weeks; 3 times per week	1RM knee extension Knee extension MVIC	1RM knee extension: significantly greater gains in strength in the high load group. Knee extension MVIC: significantly greater gains in strength in the high load group.
Jessee et al. 2018 [31]	Young untrained men (*n* = 40)	4 × 70% 1RM 4 × 15% 1RM	8 weeks; 2 times per week	1RM knee extension Isokinetic knee extension Knee extension MVIC	1RM knee extension: significantly greater gains in strength in the high load group. Isokinetic knee extension: no significant between-group differences. Knee extension MVIC: no significant between-group differences.
Kerr et al. 1996 [15]	Untrained postmenopausal middle-aged woman (*n* = 46)	3 × 8RM 3 × 20RM	1 year; 3 times per week	1RM hip extension 1RM hip flexion 1RM hip abduction 1RM hip adduction 1RM leg press 1RM wrist curl 1RM reverse wrist curl 1RM wrist pronation/supination1RM elbow flexion 1RM elbow extension	No significant between-group differences in any of the 1RM tests.
Lasevicius et al. 2018 [78]	Young untrained men (*n* = 30)	3 × 80% 1RM 3 × 60% 1RM 3 × 40% 1RM 3 × 20% 1RM	12 weeks; 2 times per week	1RM leg press 1RM elbow flexion	1RM leg press: significantly greater gains in strength in the high load (60% and 80% 1RM) groups. 1RM elbow flexion: significantly greater gains in strength in the high load (60% and 80% 1RM) groups.
Lasevicius et al. 2019 [42]	Young untrained men (*n* = 25)	3 × 80% 1RM (to failure) 3 × 80% 1RM (not to failure) 3 × 30% 1RM (to failure) 3 × 30% 1RM (not to failure)	8 weeks; 2 times per week	1RM knee extension	Significantly greater gains in strength in the high load groups.
Lim et al. 2019 [74]	Young untrained men (*n* = 21)	3 × 80% 1RM (to failure) 3 × 30% 1RM (to failure) 3 × 30% 1RM (volume—matched to 80% 1RM)	10 weeks; 3 times per week	1RM knee extension Isokinetic knee extension	1RM knee extension: no significant between-group differences. Isokinetic knee extension: significantly greater gains in strength in the low load (to failure) group.
Lopes et al. 2017 [48]	Resistance-trained men (*n* = 16)	6 × 10RM 3 × 20RM	6 weeks; 4 times per week	1RM bench press 1RM squat	1RM bench press: no significant between-group differences. 1RM squat: no significant between-group differences.
Mitchell et al. 2012 [73]	Young untrained men (*n* = 18)	3 × 80% 1RM 1 × 80% 1RM 3 × 30% 1RM	10 weeks; 3 times per week	1RM knee extension Knee extension MVIC	1RM knee extension: significantly greater gains in strength in the high load groups.Knee extension MVIC: no significant between-group differences.
Morton et al. 2016 [16]	Resistance-trained men (*n* = 49)	3 × 8–12RM 3 × 20–25RM	12 weeks; 4 times per week	1RM bench press 1RM leg press 1RM shoulder press 1RM knee extension	1RM bench press: significantly greater gains in strength in the high load group. 1RM leg press: no significant between-group differences. 1RM shoulder press: no significant between-group differences. 1RM knee extension: no significant between-group differences.
Nobrega et al. 2018 [93]	Young untrained men (*n* = 32)	3 × 80% 1RM (to failure) 3 × 80% 1RM (not to failure) 3 × 30% 1RM (to failure) 3 × 30% 1RM (not to failure)	12 weeks; 2 times per week	1RM knee extension	No significant between-group differences.
Ogasawara et al. 2013 [13]	Young untrained men (*n* = 9)	3 × 75% 1RM 4 × 30% 1RM	6 weeks; 3 times per week	1RM bench press Elbow extension MVIC	1RM bench press: significantly greater gains in strength in the high load group. Elbow extension MVIC: significantly greater gains in strength in the high load group.
Ozaki et al. 2018 [94]	Young untrained men (*n* = 9)	3 × 80% 1RM 3 × 30% 1RM	8 weeks; 2–3 times per week	1RM elbow flexion Elbow flexion MVIC	1RM elbow flexion: significantly greater gains in strength in the high load group. Elbow flexion MVIC: significantly greater gains in strength in the high load group.
Popov et al. 2006 [95]	Physically active men (*n* = 18)	3 and 7 × 80% MVIC 1 and 4 × 50% MVIC	8 weeks; 3 times per week	Knee extension MVIC	No significant between-group differences.
Rana et al. 2008 and Schuenke et al. 2012 [14,75]	Young untrained women (*n* = 27)	3 × 6–10RM 3 × 6–10RM (low velocity) 3 × 20–30RM	6 weeks; 2–3 times per week	1RM squat 1RM leg press 1RM knee extension	1RM squat: no significant between-group differences. 1RM leg press: significantly greater gains in strength in the high load group. 1RM knee extension: significantly greater gains in strength in the high load group.
Schoenfeld et al. 2015 [17]	Resistance-trained men (*n* = 18)	3 × 8–12RM 3 × 25–35RM	8 weeks; 3 times per week	1RM bench press 1RM squat	1RM bench press: no significant between-group differences. 1RM squat: significantly greater gains in strength in the high load group.
Schoenfeld et al. 2020 [26]	Young untrained men (*n* = 27)	4 × 6–10RM 4 × 20–30RM	8 weeks; 2 times per week	Ankle plantar flexion MVIC	No significant between-group differences.
Stefanaki et al. 2019 [96]	Young untrained women (*n* = 13)	1 × 80% 1RM 1 × 30% 1RM	6 weeks; 2 times per week	1RM knee extension 1RM elbow flexion	1RM knee extension: no significant between-group differences. 1RM elbow flexion: no significant between-group differences.
Stone & Coulter 1994 [6]	College-aged untrained women (*n* = 50)	3 × 6–8RM 2 × 15–20RM 1 × 30–40RM	9 weeks; 3 times per week	1RM bench press 1RM squat	1RM bench press: no significant between-group differences. 1RM squat: no significant between-group differences.
Tanimoto & Ishii 2006 [97]	Young untrained men (*n* = 24)	3 × 80% 1RM 3 × 50% 1RM (low velocity) 3 × 50% 1RM	12 weeks; 3 times per week	1RM knee extension Knee extension MVIC	1RM knee extension: no significant between-group differences. Knee extension MVIC: significantly greater gains in strength in the high load group.
Tanimoto et al. 2008 [98]	Young untrained men (*n* = 24)	3 × 80–90% 1RM 3 × 55–60% 1RM	13 weeks; 2 times per week	1RM squat 1RM chest press 1RM lat-pull down 1RM abdominal bend 1RM back extension	No significant between-group differences in any of the 1RM tests.
Van Roie et al. 2013 [29]	Young untrained males (*n* = 14) and females (*n* = 10)	1 × 10–12RM 1 × 60RM followed by 10–20RM	9 weeks; 3 times per week	1RM knee extension Knee extension MVIC Isokinetic knee extension	1RM knee extension: significantly greater gains in strength in the high load group. Knee extension MVIC: significantly greater gains in strength in the high load group. Isokinetic knee extension: significantly greater gains in strength in the low load group.
Van Roie et al. 2013 [28]	Untrained older males (*n* = 26) and females (*n* = 30)	2 × 10–15RM 1 × 80–100RM 1 × 60RM + 10–20RM	12 weeks; 3 times per week	1RM knee extension 1RM leg press Knee extension MVIC Isokinetic knee extension	1RM knee extension: significantly greater gains in strength in the high load group. 1RM leg press: significantly greater gains in strength in the high load group. Knee extension MVIC: no significant between-group differences. Isokinetic knee extension: no significant between-group differences.
Weiss et al. 1999 [99]	Young untrained men (*n* = 28)	4 × 3–5RM 4 × 13–15RM 4 × 23–25RM	7 weeks; 3 times per week	1RM squat Isokinetic knee flexion Isokinetic knee extension	1RM squat: significantly greater gains in strength in the high load group. Isokinetic knee flexion: no significant between-group differences. Isokinetic knee extension: no significant between-group differences.

RM: repetition maximum; MVIC: maximal voluntary isometric contraction; MVT: maximal voluntary torque.

**Table 2:** Summary of studies that explored the effects of different loads on muscular hypertrophy (site-specific measures).

Study	Participants	Training Groups (Sets × Load)	Training Duration; Training Frequency	Hypertrophy Outcome	Findings
Campos et al. 2002 [19]	Young untrained men (*n* = 27)	4 × 3–5RM 3 × 9–11RM 2 × 20–28RM	8 weeks; 2–3 times per week	Muscle fiber CSA (type I, IIa, and IIx)	Type I: significantly greater gains in the 3–5RM and 9–11RM groups. Type IIa: significantly greater gains in the 3–5RM and 9–11RM groups. Type IIx: significantly greater gains in the 3–5RM and 9–11RM groups.
Fink et al. 2016 [90]	Young male gymnastics athletes (*n* = 21)	3 × 8–12RM 3 × 30–40RM Mixed high and low load group: 4 weeks of 3 × 8–12RM and 4 weeks of 3 × 30–40RM	8 weeks; 3 times per week	Elbow flexor CSA	No significant between-group differences.
Fink et al. 2016 [91]	Young male gymnastics athletes (*n* = 20)	3 × 8RM 3 × 20RM	8 weeks; 3 times per week	Elbow flexor CSA	No significant between-group differences.
Hisaeda et al. 1996 [30]	Young untrained women (*n* = 11)	8–9 × 4–6RM 5–6 × 15–20RM	8 weeks; 3 times per week	Quadriceps CSA	No significant between-group differences.
Jenkins et al. 2017 [8]	Young untrained men (*n* = 26)	3 × 80% 1RM 3 × 30% 1RM	6 weeks; 3 times per week	Quadriceps muscle thickness	No significant between-group differences.
Jessee et al. 2018 [31]	Young untrained men (*n* = 40)	4 × 70% 1RM 4 × 15% 1RM	8 weeks; 2 times per week	Quadriceps muscle thickness	No significant between-group differences.
Lasevicius et al. 2018 [78]	Young untrained men (*n* = 30)	3 × 80% 1RM 3 × 60% 1RM 3 × 40% 1RM 3 × 20% 1RM	12 weeks; 2 times per week	Elbow flexor and quadriceps CSA	Elbow flexor: significantly greater gains in the 80% 1RM group compared to 20% 1RM group. Quadriceps: significantly greater gains in the 80% 1RM group compared to 20% 1RM group.
Lasevicius et al. 2019 [42]	Young untrained men (*n* = 25)	3 × 80% 1RM (to failure) 3 × 80% 1RM (not to failure) 3 × 30% 1RM (to failure) 3 × 30% 1RM (not to failure)	8 weeks; 2 times per week	Quadriceps CSA	Significantly greater gains in the 80% 1RM groups and the 30% 1RM group (to failure)
Lim et al. 2019 [74]	Young untrained men (*n* = 21)	3 × 80% 1RM (to failure) 3 × 30% 1RM (to failure) 3 × 30% 1RM (volume-matched to 80% 1RM)	10 weeks; 3 times per week	Muscle fiber CSA (type I and type II)	Type I: significantly greater gains in the groups training to failure. Type II: no significant between-group differences.
Mitchell et al. 2012 [73]	Young untrained men (*n* = 18)	3 × 80% 1RM 1 × 80% 1RM 3 × 30% 1RM	10 weeks; 3 times per week	Quadriceps CSA, muscle fiber CSA (type I and type II)	No significant between-group differences in any of the outcomes.
Morton et al. 2016 [16]	Resistance-trained men (*n* = 49)	3 × 8–12RM 3 × 20–25RM	12 weeks; 4 times per week	Muscle fiber CSA (type I and type II)	No significant between-group differences in any of the outcomes.
Nobrega et al. 2018 [93]	Young untrained men (*n* = 32)	3 × 80% 1RM (to failure) 3 × 80% 1RM (not to failure) 3 × 30% 1RM (to failure) 3 × 30% 1RM (not to failure)	12 weeks; 2 times per week	Quadriceps CSA	No significant between-group differences.
Ogasawara et al. 2013 [13]	Young untrained men (*n* = 9)	3 × 75% 1RM 4 × 30% 1RM	6 weeks; 3 times per week	Triceps brachii and pectoralis major CSA	Triceps brachii: no significant between-group differences. Pectoralis major: no significant between-group differences.
Ozaki et al. 2018 [94]	Young untrained men (*n* = 9)	3 × 80% 1RM 3 × 30% 1RM	8 weeks; 2–3 times per week	Elbow flexor CSA	No significant between-group differences.
Popov et al. 2006 [95]	Physically active men (*n* = 18)	3 and 7 × 80% MVIC 1 and 4 × 50% MVIC	8 weeks; 3 times per week	Quadriceps and gluteus CSA	Quadriceps: no significant between-group differences. Gluteus: no significant between-group differences.
Rana et al. 2008 and Schuenke et al. 2012 [14,75]	Young untrained women (*n* = 27)	3 × 6–10RM 3 × 6–10RM (low velocity) 3 × 20–30RM	6 weeks; 2–3 times per week	Muscle fiber CSA (type I, IIa and IIx)	Type I: significantly greater gains in the high load group. Type IIa: significantly greater gains in both high load groups. Type IIx: significantly greater gains in both high load groups.
Schoenfeld et al. 2015 [17]	Resistance-trained men (*n* = 18)	3 × 8–12RM 3 × 25–35RM	8 weeks; 3 times per week	Elbow flexor, elbow extensor, and quadriceps muscle thickness	No significant between-group differences in any of the outcomes.
Schoenfeld et al. 2020 [26]	Young untrained men (*n* = 27)	4 × 6–10RM 4 × 20–30RM	8 weeks; 2 times per week	Calf muscle thickness	No significant between-group differences.
Stefanaki et al. 2019 [96]	Young untrained women (*n* = 13)	1 × 80% 1RM 1 × 30% 1RM	6 weeks; 2 times per week	Elbow flexor and quadriceps muscle thickness	Elbow flexor: no significant between-group differences. Quadriceps: no significant between-group differences.
Tanimoto & Ishii 2006 [97]	Young untrained men (*n* = 24)	3 × 80% 1RM 3 × 50% 1RM (low velocity) 3 × 50% 1RM	12 weeks; 3 times per week	Quadriceps CSA	Significantly greater gains in the 80% 1RM and 50% 1RM (low velocity) groups.
Tanimoto et al. 2008 [98]	Young untrained men (*n* = 24)	3 × 80–90% 1RM 3 × 55–60% 1RM	13 weeks; 2 times per week	Upper and lower-body muscle thickness (multiple sites)	No significant between-group differences.
Van Roie et al. 2013 [28]	Untrained older males (*n* = 26) and females (*n* = 30)	2 × 10–15RM 1 × 80–100RM 1 × 60RM + 10–20RM	12 weeks; 3 times per week	Quadriceps CSA	No significant between-group differences.

RM: repetition maximum; CSA: cross-sectional area.

**Table 3:** Summary of studies that explored the effects of different loads on muscular endurance.

Study	Participants	Training Groups (Sets × Load)	Training Duration; Training Frequency	Muscular Endurance Test	Findings
Anderson et al. 1982 [5]	Male college students (*n* = 43)	3 × 6–8RM 2 × 30–40RM 1 × 100–150RM	9 weeks; 3 times per week	Relative endurance: 40% 1RM (post-intervention values) in the bench press Absolute endurance: 27 kg in the bench press	Relative endurance: significantly greater gains in muscular endurance in the low load groups. Absolute endurance: no significant between-group differences.
Campos et al. 2002 [19]	Young untrained men (*n* = 27)	4 × 3–5RM 3 × 9–11RM 2 × 20–28RM	8 weeks; 2–3 times per week	Relative endurance: 60% 1RM (post-intervention values) in the squat, leg press, and knee extension	Squat: significantly greater gains in muscular endurance in the low load group. Leg press: significantly greater gains in muscular endurance in the low load group. Knee extension: significantly greater gains in muscular endurance in the low load group.
Jessee et al. 2018 [31]	Young untrained men (*n* = 40)	4 × 70% 1RM 4 × 15% 1RM	8 weeks; 2 times per week	Relative endurance: 42.5% 1RM (pre-intervention values) in the knee extension	No significant between-group differences.
Mitchell et al. 2012 [73]	Young untrained men (*n* = 18)	3 × 80% 1RM 1 × 80% 1RM 3 × 30% 1RM	10 weeks; 3 times per week	Relative endurance: 30% and 80% 1RM (post-intervention values) in the knee extension	80% 1RM: no significant between-group differences. 30% 1RM: significantly greater gains in muscular endurance in the low load group.
Ozaki et al. 2018 [94]	Young untrained men (*n* = 9)	3 × 80% 1RM 3 × 30% 1RM	8 weeks; 2-3 times per week	Relative endurance: 30% 1RM (post-intervention values) in the elbow flexion	Significantly greater gains in muscular endurance in the low load group.
Rana et al. 2008 and Schuenke et al. 2012 [14,75]	Young untrained women (*n* = 27)	3 × 6–10RM 3 × 6–10RM (low velocity) 3 × 20–30RM	6 weeks; 2–3 times per week	Relative endurance: 60% 1RM (post-intervention values) in the squat, leg press, and knee extension	Squat: no significant between-group differences. Leg press: no significant between-group differences. Knee extension: no significant between-group differences.
Schoenfeld et al. 2015 [17]	Resistance-trained men (*n* = 18)	3 × 8–12RM 3 × 25–35RM	8 weeks; 3 times per week	Relative endurance: 50% 1RM (post-intervention values) in the bench press	Significantly greater gains in muscular endurance in the low load group.
Stone & Coulter 1994 [6]	College-aged untrained women (*n* = 50)	3 × 6–8RM 2 × 15–20RM 1 × 30–40RM	9 weeks; 3 times per week	Relative endurance: 45% 1RM (pre-intervention values) in the bench press 45% 1RM (post-intervention values) in the bench press 55% 1RM (pre-intervention values) in the squat 55% 1RM (post-intervention values) in the squat Absolute endurance: 16 kg in the bench press 16 kg in the squat	Relative endurance (pre-intervention values): no significant between-group differences in the bench press or squat. Relative endurance (post-intervention values): no significant between-group differences in the bench press. Significantly greater gains in muscular endurance in the 6–8RM and 30–40RM groups. Absolute endurance: no significant between-group differences in the bench press or squat.
Van Roie et al. 2013 [29]	Young untrained males (*n* = 14) and females (*n* = 10)	1 × 10–12RM 1 × 60RM followed by 10–20RM	9 weeks; 3 times per week	Isokinetic knee extension maximum work	Significantly greater gains in muscular endurance in the low load group.
Van Roie et al. 2013 [28]	Untrained older males (*n* = 26) and females (*n* = 30)	2 × 10–15RM 1 × 80–100RM 1 × 60RM + 10–20RM	12 weeks; 3 times per week	Relative endurance: 60% 1RM (post-intervention values) in the knee extension	No significant between-group differences.

RM: repetition maximum; MVIC: maximal voluntary isometric contraction; MVT: maximal voluntary torque.

## Data Availability

No new data were created or analyzed in this study. Data sharing is not applicable to this article.
